# Supplementary material for: The UV–Vis spectrum of the ClCO radical in the catalytic cycle of Cl-initiated CO oxidation
Source: Commun Chem. 2025 May 24;8:163. doi: 10.1038/s42004-025-01520-3 (PMC12103620; doi:10.1038/s42004-025-01520-3)
Supplement: Supplementary file 2 — Supplementary Information [file 42004_2025_1520_MOESM2_ESM.pdf]

The UV–Vis Spectrum of the ClCO Radical in the Catalytic Cycle of the Cl-Initiated CO Oxidation

Wen Chao,[1] Robert Skog,[3] Benjamin N. Frandsen,[3,4] Gregory H. Jones,[1,†] Kayla T. Pham,[7] Mitchio Okumura,[1] Mads P. Sulbaek Andersen,[5,6] Carl J. Percival,[2] Frank A. F. Winiberg[2,*]

[1] Division of Chemistry and Chemical Engineering, California Institute of Technology, 1200 E California Blvd, Pasadena, CA 91125 United States

[2] Jet Propulsion Laboratory, California Institute of Technology, 4800 Oak Grove Drive, Pasadena, CA 91109-8099, United States

[3] Department of Chemistry, University of Helsinki, P. O. Box 55 (A. I. Virtasen aukio 1), FI-00014 Helsinki, Finland.

[4] Aerosol Physics Laboratory, Tampere University, Korkeakoulunkatu 3, FI-33720 Tampere, Finland.

[5] Department of Chemistry and Biochemistry, California State University Northridge, Northridge, CA 91330-8262, United States

[6] Copenhagen Center for Atmospheric Research, Department of Chemistry, University of Copenhagen, Universitertsparken 5, DK-2100 Copenhagen Ø, Denmark

[7] Department of Chemistry, Columbia University, New York, New York, 10027, United States

† Now as postdoc at Department of Chemistry, University of Florida, Gainesville, Florida 32611, United States

*Email: fred.a.winiberg@jpl.nasa.gov

Table of Contents

Error Analysis S2

The vibrational progressions of A-band spectrum (Fig. S1–S2) S4

The $Cl_2SO/CO/N_2$/248 nm photolysis system (Fig. S3) S5

Details of the Orbital Coefficients of Vertical Transitions to Excited States (Table S1–S2) S6

Comparisons with HCO and FCO S8

Details of the HEAT calculations (Table S3) S9

Effects of basis set and temperature on the simulated spectrum (Fig. S4–S5) S10

References S11

Error Analysis

The error of all the reported values was estimated assuming the uncertainties from all the potential factors are uncorrelated.

In detail, the CO flow was controlled by a MKS 1179A series mass flow controller (MFC) and the N_2 flow was controlled by one MKS 1179A series and one MKS GM series MFC for matching the total mass flow rate to the gas refresh time. The 1179A and GM series MFC have accuracy about 5% and 0.2% after calibration. The MKS 127 AA series pressure gauges have an overall accuracy of 0.5%. However, our pressure gauge shows a baseline of 8 Torr with a fluctuation of 1 Torr at vacuum, which gives an accuracy of 1 Torr in our experiments. The Cl_2 mass flow has 1% error after calibration. The CO concentrations were derived from the flows and the pressures in the reactor as shown below, where P_{CO} is the CO partial pressure, P_{total} is the total pressure, F_{CO}, F_{N2} and F_{Cl2} is the flow of CO, N_2, and Cl_2 gases.

$$P_{CO} = P_{total} \frac{F_{CO}}{F_{CO} + F_{N2} + F_{Cl}}$$

Thus, we estimated the error in CO concentration using the formula below.

$$\delta_{[CO]} = 0.088 = \sqrt{0.01^2 + 2 \times 0.05^2 + 0.05^2 + 0.01^2} = \sqrt{\delta_P + 2 \times \delta_{FCO} + \delta_{FN2} + \delta_{FCl2}}$$

The error of the equilibrium constant primarily comes from the CO concentration and the precision of the collected data, which is estimated from the error in the fitting process, ~8.1%. Therefore, the estimated error in the ClCO equilibrium constant is 12% for one standard deviation.

$$\delta_{K\_ClCO} = 0.12 = \sqrt{0.081^2 + 0.088^2} = \sqrt{\delta_{K\_fit} + \delta_{[CO]}}$$

The normalization of CO dependence will not affect the determination of the equilibrium constant.

The error in the absolute ClCO cross section is primarily dominated by the estimation of the initial Cl concentration, $[Cl]_0$. We estimated a baseline stability in our system about 5×10^{-4} in absorbance. The measured Cl_2 depletion signals are about 5×10^{-3}, which results in a relative error of 10%. The effective

absorption length was characterized previously by monitoring the absorption signals of NO_2, which gives an average error about δ_L = 10%. The fitting of the CO dependence (Figure 2C, main text) yielded a precision of ~2 %. The absolute cross section of Cl_2 has been measured by many groups and their consistency is about 0.5%.[1] Considering all the factor above, the error of the absolute cross section is δ_σ = 14% of one standard deviation.

To the best of our knowledge, the reaction rate coefficients of the ClCO self-reaction is still unknown. The weak Cl–CO bond implies a fast reaction between the ClCO radical. It is hard to estimate the loss of Cl atoms through the formation of the ClCO radical. As a result, the estimated absolute cross section reported in this work should be treated as a lower bound and no uncertainty is shown in the main text.

The vibrational progressions of A-band spectrum

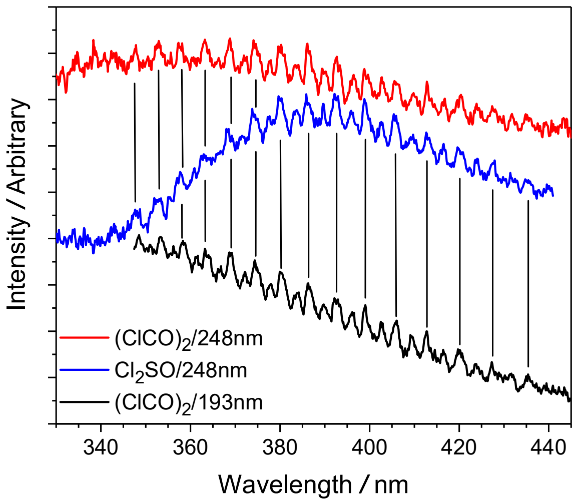

Figure S1. Spectra recorded using a 600 grooves/mm grating for different chlorine atom precursors and different photolysis wavelengths shows the same vibrational progression near 380 nm.

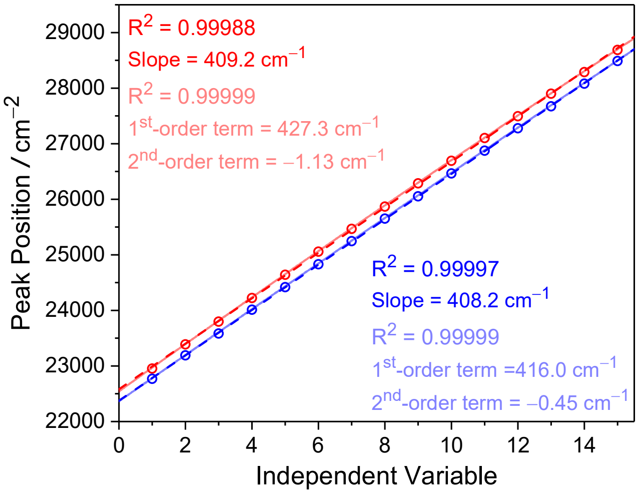

Figure S2. Linear and quadratic fits of the observed positions of the vibrational progressions. Red circles represent the strong series indicated in Figure S1.

The $Cl_2SO/CO/N_2$/248 nm photolysis system

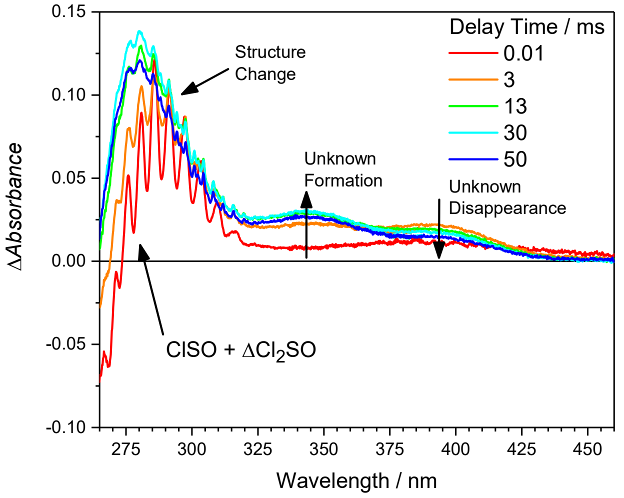

Figure S3. Representative spectra of $Cl_2SO/CO/N_2$ mixture photolysis at 248 nm at 238 K without oxygen. The experimental conditions are $[Cl_2SO] = 1.2\times10^{15}$ cm^{-3}, P_{CO} = 6.2 Torr, and P_{total} = 50 Torr. The strong absorption from unknown species causes low baseline stability for small absorption signals.

Details of the Orbital Coefficients of Vertical Transitions to Excited States

Table S1. Individual contributions of the orbital coefficients to the A' excited states. The (I, j, A, b) means excitation from occupied alpha I and beta j orbitals to virtual alpha A and beta b orbitals. Note that alpha 16 orbital is the SOMO.

$1^2A' \leftarrow X^2A'$						$2^2A' \leftarrow X^2A'$					
**I**	**j**	**A**	**b**	**Coe.**	**Spin**	**I**	**j**	**A**	**b**	**Coe.**	**Spin**
0	15	0	16	0.910	BB	16	0	18	0	-0.796	AA
0	15	0	18	0.143	BB	0	13	0	16	0.317	BB
0	15	0	24	0.097	BB	16	0	19	0	-0.147	AA
16	0	18	0	-0.091	AA	0	11	0	16	-0.146	BB
0	15	0	20	-0.087	BB	16	0	21	0	0.139	AA
16	15	18	16	0.070	ABAB	16	0	24	0	0.133	AA
0	15	0	29	-0.052	BB	16	0	20	0	0.126	AA
13	15	17	16	-0.046	ABAB	15	0	17	0	0.114	AA
0	13	0	18	0.046	BB	0	14	0	17	0.100	BB
15	0	17	0	-0.045	AA	13	0	17	0	0.089	AA
11	15	16	18	0.044	BBBB	12	0	18	0	-0.072	AA
13	0	17	0	-0.044	AA	16	0	23	0	0.071	AA
16	13	18	16	0.043	ABAB	12	13	18	16	0.071	ABAB
16	15	17	17	-0.043	ABAB	16	0	34	0	0.061	AA
16	15	21	16	-0.038	ABAB	16	0	27	0	0.059	AA

Table S2. Individual contributions of the orbital coefficients to the A" excited states. The (I, j, A, b) means excitation from occupied alpha I and beta j orbitals to virtual alpha A and beta b orbitals. Note that alpha 16 orbital is the SOMO.

$1^2A'' \leftarrow X^2A'$						$2^2A'' \leftarrow X^2A'$					
**I**	**j**	**A**	**b**	**Coe.**	**Spin**	**I**	**j**	**A**	**b**	**Coe.**	**Spin**
16	0	17	0	0.922	AA	0	14	0	16	-0.916	BB
11	0	17	0	0.129	AA	0	14	0	18	-0.150	BB
16	0	31	0	-0.115	AA	0	14	0	24	-0.099	BB
14	0	17	0	-0.080	AA	0	14	0	20	0.088	BB
12	13	17	16	-0.080	ABAB	16	14	18	16	-0.082	ABAB
16	0	22	0	0.071	AA	0	14	0	29	0.051	BB
12	0	17	0	0.060	AA	0	12	0	18	0.050	BB
0	13	0	17	0.057	BB	11	14	16	18	-0.049	BBBB
10	0	17	0	-0.056	AA	13	14	17	16	0.047	ABAB
10	13	17	16	0.055	ABAB	16	14	17	17	0.043	ABAB
0	12	0	16	-0.054	BB	15	0	18	0	0.043	AA
12	12	17	17	0.049	ABAB	13	12	17	16	0.040	ABAB
16	0	28	0	-0.047	AA	16	14	21	16	0.039	ABAB
11	11	17	16	0.042	ABAB	16	14	24	16	0.039	ABAB
16	11	17	16	-0.037	ABAB	16	14	19	16	-0.038	ABAB

Comparison with the HCO and FCO

We anticipated that the ClCO radical shares a similar electronic structure with its analogs, HCO and FCO, which exhibit clear vibronic progression for the A←X transition between the Renner-Teller pairs. For HCO and FCO radicals, the observed vibronic spacing of the transition between Renner-Teller pairs is approximately twice that of the theoretical prediction,[2,3] contrasting with the observations and predictions for the ClCO radical case. This discrepancy could be rationalized by differing excited state lifetimes due to the Renner-Teller effect, which posits that only vibronic states with zero vibrational angular momentum exhibit long enough lifetimes to show sharp peaks. This is not a strict selection rule as broad and diffused bands have been observed for HCO and FCO.[2,3] The conical intersection at linear ClCO structure opens a pathway to couple to the repulsive state beyond the Renner-Teller pair, broadening every vibronic transition regardless of the vibrational angular momentum. Consequently, the spectrum profile with alternating diffuse and sharp peaks disappears, and smaller absorption cross sections are expected.

The mixing of three states and the significant structural change for the $1^2A''$ state pose challenges in simulating the vibronic progression. As a first-order approximation, we simulated the observed spectrum of ClCO by sampling the ground state geometry for the overall band shape and the absorption cross section. High-resolution transition linewidth measurements could yield further spectroscopic insights but is beyond the scope of the present work.

Details of the HEAT calculations

Table S3. Individual contributions to the HEAT total energies (in Hartree) and the standard enthalpy change at 0 K.

Species	E^{∞}_{HF}	$\Delta E^{\infty}_{CCSD(T)}$	ΔE_{CCSDT}	ΔE_{CCSDTQ}	ΔE_{REL}	ΔE_{DBOC}	ΔE_{SO} [a]	ΔE_{ZPE}	Total
Cl	-459.489895	-0.665245	-0.000767	-0.000161	-1.404007	0.005940	-0.001338	0.000000	-461.555473
CO	-112.790997	-0.535543	0.000097	-0.000951	-0.067285	0.003999	0.000000	0.004946	-113.385735
Cl_2	-919.010527	-1.395247	-0.000841	-0.000707	-2.807659	0.011876	0.000000	0.001264	-923.201840
ClCO	-572.259204	-1.235703	-0.000513	-0.001413	-1.471009	0.009955	0.000000	0.006536	-574.951351
Cl_2CO	-1031.822703	-1.954091	-0.000211	-0.001783	-2.874534	0.015840	0.000000	0.010569	-1036.626913

Reaction	$\Delta H°$(0 K, HEAT) / kJ mol^{-1} [a]	$\Delta H°$(0 K, HEAT) / kcal mol^{-1} [b]
ClCO → Cl + CO	26.63	6.36
ClCO + Cl → Cl_2 + CO	-212.0	-50.67
ClCO + Cl → Cl_2CO	-315.3	-75.36
ClCO + ClCO → Cl_2 + 2CO	-185.4	-44.31
ClCO + ClCO → Cl_2CO + CO	-288.7	-68.99

[a] 1 Hartree = 2625.4976 kJ mol^{-1}

[b] 1 kcal = 4.184 kJ

Effects of Basis Set and Temperature on the Simulated Spectra

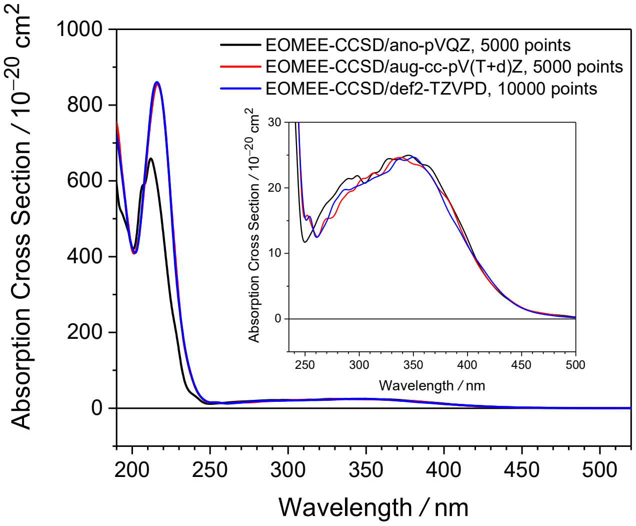

Figure S4. The simulated ClCO spectrum at EOMEE-CCSD level with ano-pVQZ (black), aug-cc-pV(T+d)Z (red) and def2-TZVPD (blue) basis sets at 0 K.

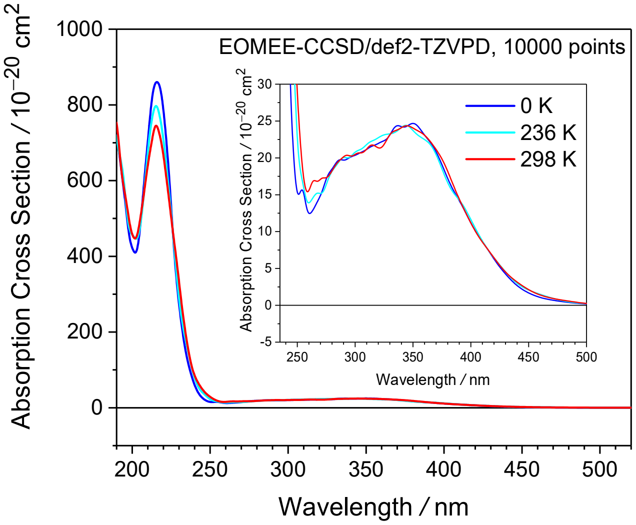

Figure S5. The simulated ClCO spectrum, sampling 10000 geometries, at EOMEE-CCSD/def2-TZVPD level at 0 K, 236 K, and 298 K.

References

1. Burkholder, J. B. *et al.* JPL Publication 19-5. Chemical Kinetics and Photochemical Data for Use in Atmospheric Studies. *JPL Publication* **19–5**, 2–7 (2020).
2. Ndengué, S. A., Dawes, R. & Guo, H. A new set of potential energy surfaces for HCO: Influence of Renner-Teller coupling on the bound and resonance vibrational states. *J. Chem. Phys.* **144**, 244301 (2016).
3. Howie, W. H., Lane, I. C. & Orr-Ewing, A. J. The near ultraviolet spectrum of the FCO radical: Re-assignment of transitions and predissociation of the electronically excited state. *The Journal of Chemical Physics* **113**, 7237–7251 (2000).
